# An integrated primary care-based programme of PRE-Pregnancy cARE to improve pregnancy outcomes in women with type 2 Diabetes (The PREPARED study): protocol for a multi-method study of implementation, system adaptation and performance

**DOI:** 10.1186/s12875-022-01683-1

**Published:** 2022-04-13

**Authors:** Rita Forde, Olubunmi Abiola, Janet Anderson, Debra Bick, Anna Brackenridge, Anita Banerjee, Mark Chamley, Kia-Chong Chua, Lily Hopkins, Katharine Hunt, Helen R. Murphy, Helen Rogers, Renee Romeo, James Shearer, Kirsty Winkley, Angus Forbes

**Affiliations:** 1 Faculty of Nursing, Midwifery and Palliative Care, King’s College London, James Clerk Maxwell Building, 57 Waterloo Road, London, UK; 2 PPI Member, c/o Faculty of Nursing, Midwifery and Palliative Care, King’s College London, James Clerk Maxwell Building, 57 Waterloo Road, London, UK; 3 School of Health Sciences, City, University of London, Northampton Square, London, UK; 4 Warwick Clinical Trials Unit, Warwick Medical School, University of Warwick, Gibbet Hill, Coventry, UK; 5 Diabetes and Endocrinology Department, Guy’s and St Thomas’ Hospital NHS Foundation Trust, London, UK; 6 North Wood Group Practice, Crown Dale, Norwood, London, UK; 7 Centre for Implementation Science, Institute of Psychiatry, Psychology and Neuroscience, King’s College London, London, SE5 8AF UK; 8 Diabetes Department, King’s College Hospital NHS Foundation Trust, Caldecot Road, London, UK; 9 Norwich Medical School, University of East Anglia, Norwich Research Park, Norwich, UK; 10 Health Services and Population Research, Institute of Psychiatry, Psychology and Neuroscience, King’s College London, London, UK

**Keywords:** Pre-pregnancy care, Type 2 diabetes, Complex adaptive systems, Primary care

## Abstract

**Background:**

The number of women of childbearing age with Type 2 diabetes(T2DM) is increasing, and they now account for > 50% of pregnancies in women with pre-existing diabetes. Diabetes pregnancies without adequate pre-pregnancy care have higher risk for poor outcomes (miscarriages, birth-defects, stillbirths) and are associated with increased complications (caesarean deliveries, macrosomic babies, neonatal intensive-care admissions). The risks and costs of these pregnancies can be reduced with pregnancy preparation (HbA1c, ≤ 6.5%, 5 mg folic acid and stopping potentially harmful medicines). However, 90% of women with T2DM, most of whom are based in primary care, are not adequately prepared for pregnancy. This study will evaluate a programme of primary care-based interventions (decision-support systems; pre-pregnancy care-pathways; pregnancy-awareness resources; professional training; and performance monitoring) to improve pregnancy preparation in women with T2DM.

**Methods:**

The study aims to optimise the programme interventions and estimate their impact on pregnancy preparation, pre-pregnancy care uptake and pregnancy outcomes. To evaluate this multimodal intervention, we will use a multi-method research design following Complex Adaptive Systems (CAS) theory, refining the interventions iteratively during the study. Thirty GP practices with ≥ 25 women with T2DM of reproductive age (18–45 years) from two South London boroughs will be exposed to the intervention. This will provide > 750 women with an estimated pregnancy incidence of 80–100 to study. The research involves: a clinical audit of processes and outcomes; a process evaluation informing intervention feasibility, implementation, and behaviour change; and a cost-consequences analysis informing future economic evaluation. Performance data will be collected via audits of GP systems, hospital antenatal clinics and pregnancy outcomes. Following CAS theory, we will use repeated measurements to monitor intervention impact on pregnancy preparation markers at 4-monthly intervals over 18-months. We will use performance and feasibility data to optimise intervention effects iteratively. The target performance for the intervention is a 30% increase in the proportion of women meeting pre-pregnancy care criteria.

**Discussion:**

The primary output will be development of an integrated programme of interventions to improve pregnancy preparation, pre-pregnancy care uptake, and reduce adverse pregnancy outcomes in women with T2DM. We will also develop an implementation plan to support the introduction of the interventions across the NHS.

**Trial registration:**

ISRCTN47576591; February 8, 2022.

## Background

As the prevalence of Type 2 diabetes (T2DM) continues to increase [1], the age of onset has also been reducing [2]. Consequently there are now many more women of reproductive age living with T2DM, with half of pregnancies in women with pre-existing diabetes in England and Wales now occurring in women with T2DM [3]. This proportion is greater in areas such as London where 70% of pregnancies are in women with T2DM [3], due to the higher numbers of women of Black or Asian ethnicity as these populations tend to have an earlier age of onset [2, 4]. Diabetes pregnancies are associated with multiple hazards and higher care costs. Compared to women without diabetes, women with diabetes have: double the risks for congenital abnormality or stillbirth [5–10] and a quadrupled risk of fetal death [5, 6]. Concerningly, these adverse outcomes are increasing, with no progress having been made in the last two decades [11]. These risks are now more evident in women with T2DM, as the proportion of stillbirths and neonatal deaths is higher in women with T2DM compared to those with Type 1 diabetes (T1DM) [3, 5]. The most recent National Pregnancy In Diabetes (NPID) audit of pregnancy outcomes in women with T2DM reported that: 26% of pregnancies resulted in large for gestational age (LGA) babies; 50% of births were by caesarean section; and 15% of infants are admitted to neonatal care [3, 12]. Hence, diabetes pregnancies in women with T2DM are increasing, these are high risk, currently have poor pregnancy outcomes and are associated with increased treatment costs.

Many of the risks associated with diabetes pregnancies can be ameliorated with improved pregnancy preparation and specific pre-pregnancy care clinic attendance. It is well established that exposure to hyperglycaemia and some treatments prescribed in the management of T2DM can harm the fetus during the period of organogenesis in the first trimester [13, 14]. Unfortunately, many women with T2DM are unaware of these risks [15] and may not realise they have conceived until late into the first trimester of their pregnancy [16]. In response, the National Institute for Health and Care Excellence (NICE) has developed guidelines for pre-pregnancy care (PPC) in women with diabetes which recommends: initiating high-dose (5 mg) folic acid; avoiding teratogenic medications; and intensifying glucose control [17]. However, the uptake of PPC is very low in the UK, with only 10% of women with T2DM meeting the current NICE criteria [3] increasing the risk of adverse pregnancy outcomes [18, 19]. It is also concerning that the majority of these women are from deprived areas and are from Black or Asian minority populations, thereby heightening underlying health inequalities [3]. Hence, establishing effective interventions to improve the uptake of PPC in women with T2DM is a high priority.

Previous prospective cohort studies investigating interventions to improve PPC uptake in women with diabetes have had a limited impact among women with T2DM, increasing uptake by only 7–15% [20–23]. These interventions have included: health professional education; patient information and education on PPC; PPC teams; and clinical guidelines [20–23].

In the most recent UK study, while there was a significant improvement in 'optimal' pregnancy preparation among women with T2DM (5.8% and 15.1%; *p* = 0.021), defined as having HbA1c ≤ 48 mmol/mol (6.5%), on folic acid 5 mg daily and not taking harmful medications prior to last menstrual period, and attend antenatal care at ≤ 8 weeks gestation, the majority of this group (85%) were not optimally prepared for pregnancy, with no significant changes in teratogenic medicine exposure (16% before and 12.2% during/after PPC implementation; *p* = 0.41) [23]. Limitations of these studies included: a failure to consider the behavioural factors that mediate PPC uptake in the context of women with T2DM, health professionals or the care system; and that they focussed on both women with T1DM and T2DM where the care contexts are quite divergent. In women with T2DM it is important to integrate PPC into primary care as this is where most women receive diabetes support, in contrast to those with T1DM who are managed in specialist services, in the UK. In addition, it is also important to consider women’s cultural beliefs and context as these can have a powerful influence on their reproductive health behaviours [15]. Therefore, if the uptake of PPC in women with T2DM is to be improved a new approach is required. Hence, we have developed an intervention programme that is exclusive to women with T2DM and has been theoretically modelled to specific target behaviours with a focus on primary care; this programme is called PREPARED (**PRE-P**regnancy c**ARE** for women with type 2 **D**iabetes).

## Development of PREPARED

**Fig. 1:**
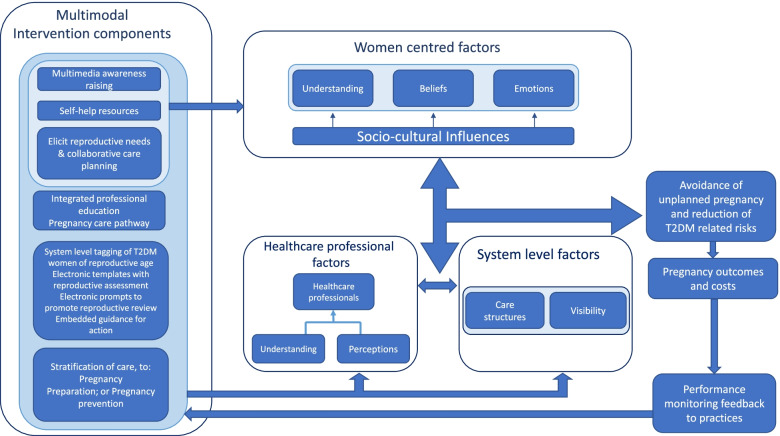
Integrating areas of potential intervention

The PREPARED programme is comprised of an integrated portfolio of interventions developed following the Medical Research Council (MRC) complex interventions framework [24], based on preliminary theory and modelling work that included: a systematic review of previous interventions [25]; a meta-synthesis of women’s experiences of PPC [26]; and a qualitative study with women with T2DM (*n* = 30) and health professionals (*n*= 22) exploring experiences of PPC and how to improve PPC uptake [15, 27]. The data from this work were used to develop a logic model mapping the key factors that mediate PPC uptake in relation to the women, health professionals and the care system. The intervention components were then integrated into the model targeting different mediating factors (see Fig. 1). As many of the mediating factors were behavioural or related to care systems, we also modelled the programme using: the COM-B (capability, opportunity, motivation to perform a behaviour) and the Behaviour Change Wheel (BCW) frameworks [28]; and Normalisation Process Theory [29, 30].

Throughout the development of the intervention, we have also been working with a group of women with T2DM who have experienced a diabetes pregnancy. Together with them and health professionals from primary care, diabetes services and maternity care we have built the PREPARED intervention, which is comprised of the following theoretically modelled components:

- decision support systems to prompt pregnancy review in primary care;
- an integrated primary care care-pathway for PPC linking to diabetes intermediate care teams, women’s health and maternity services;
- reproductive intention and pregnancy reviews with collaborative care-planning for all eligible women;
- materials for promoting pregnancy awareness in women with T2DM;
- web-based information resources for women;
- and performance monitoring with feedback to practices on PPC activity and pregnancy outcomes.

The most important feature of our approach is that it is centred in primary care where the women receive their routine diabetes management, rather than hospitals where most PPC is currently provided. PREPARED involves installing interventions to promote PPC within the electronic management systems of general practices in primary care, along with a training package for the practice team. In this paper we outline the protocol for a study which will aim to optimise the different components of the PREPARED intervention by assessing their impact on women’s access to PPC (or contraception) and pregnancy outcomes. The study will start in October 2021 and is funded by the UK government through the Health Services and Delivery Research programme of the National Institute of Health Research (NIHR131250).

## Methods

### Study approach

Reflecting the multimodal nature of the PREPARED programme and the complex care context for our study, we have designed the study following Complex Adaptive Systems (CAS) theory. CAS is an emerging model for evaluating complex interventions that considers: interdependencies within the studied system; the role of learning; interactions in self-organisation (stable/dynamic) among system components; emergence, the way different intervention components might interact leading to unexpected outcomes; and co-evolution, the way the system adapts to the intervention components and vice versa [31]. CAS theory allows interventions to be developed iteratively by boosting or optimising intervention components based on performance during the evaluation period. For this study we will use a blended approach based on complexity and implementation science, as advocated by Braithwaite et al. (2018); [32] with an integrated process evaluation following the MRC process evaluation model for complex interventions [33]. To address the implementational element within the study we have used Normalisation Process Theory (NPT) [29, 30] to study the performance and adoption of the PREPARED intervention, considering: coherence (differentiation, specification and internalisation); cognitive participation (initiation, enrolment and activation); collective action (workability and integration); and reflexive monitoring (systematisation and appraisal). A preliminary economic evaluation in the form of a cost consequences analysis will summarise costs and outcomes to inform future definitive evaluations of cost-effectiveness of the PREPARED intervention.

### Study objectives

The study aims to optimise the PREPARED intervention components and to estimate their impact on the uptake of PPC and pregnancy outcomes. The study objectives are to:

- observe the impact of the intervention components and iteratively optimise their effect on care processes and outcomes;
- assess intervention feasibility (acceptability, utility, reach, and fidelity);
- understand the organisational factors that mediate intervention implementation and performance;
- study the impacts of intervention components on the behaviours of health professionals and women with T2DM;
- generate theoretical models for implementing PPC;
- and undertake preliminary estimation of intervention costs and benefits.

### Study design

A multimethod design will be used, with a clinical audit of PPC related processes and outcomes; and an integrated process evaluation informing intervention feasibility, implementation, and behaviour change; and an economic cost consequences analysis. The clinical audit will provide PPC performance data. Following CAS theory, we will use frequent repeat measures of the PPC performance metrics to monitor the delivery and impact of the intervention components at four-monthly intervals over 18 months from baseline (performance 12 months pre-intervention). At each timepoint performance measures will be reviewed to inform where intervention effects might need boosting or refining. The target performance for the intervention will be a minimum improvement of 30% at final observation in the proportion of women meeting current NICE pregnancy criteria [17]. Practice level performance will also be monitored considering the number of women: having a reproductive review; accessing PPC; receiving folic acid 5 mg; and instigated on contraception. Table 1 provides an overview of the data that will be used to determine systemic (across the exposed practices) and practice level performance (see Table 1). The PPC and pregnancy outcomes will have a relatively low incidence at the practice level so these will only be considered at the systemic level.

**Table 1:** Performance measures

Performance measures	Systemic Performance	Practice Performance
Process data		
• Reproductive review and care plan instigated	Yes	Yes
• Care pathway activation	Yes	Yes
• Initiation of folic acid 5 mg	Yes	Yes
• Contraception advise given	Yes	Yes
• Instigated on contraception	Yes	Yes
• HbA1c testing and results	Yes	Yes
Pre-pregnancy care targets		
• HbA1c ≤ 48 mmol/mol (6.5%)	Yes	No
• Prescription of 5 mg folic acid pre-pregnancy	Yes	No
• Cessation of potentially teratogenic therapies	Yes	No
Pregnancy outcomes		
• Neonatal hypoglycaemia	Yes	No
• Macrosomia (birth weight > 4.5 kg)	Yes	No
• Shoulder dystocia	Yes	No
• Caesarean section incidence	Yes	No
• Neonatal intensive care admissions	Yes	No
• Postnatal length of stay	Yes	No
• Any other adverse outcomes	Yes	No

**Fig. 2:**
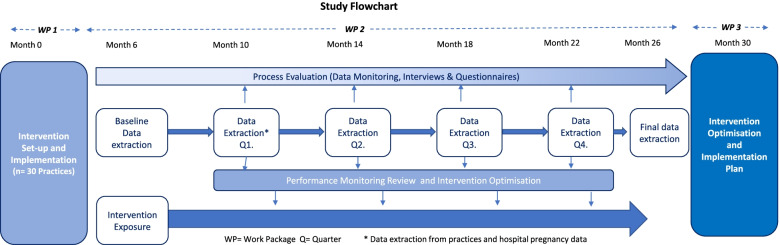
Study flowchart

The process evaluation will assess: the reach, fidelity, delivery and receipt of the programme; the women’s and health professionals’ experiences of the interventions; mechanisms of action, including changes in women’s and health professionals’ behaviours; and system level interactions. Data will also be collected on how the intervention affects the ability of clinicians to anticipate and respond to women’s needs, monitor performance, and learn from positive and negative outcomes, which are key characteristics of adaptive systems [34, 35]. The process evaluation will use a mixed-method approach incorporating both qualitative and quantitative data which will be collected at multiple time points to provide opportunities for data triangulation and subsequent theory generation [33]. An overview of the study design is presented schematically in Fig. 2.

### Study setting, sample size and participants

The study will take place in South London where currently a high proportion of women with T2DM become pregnant without effective PPC [3]. We will recruit 30 general practices for intervention exposure. Practices will be considered for inclusion if they have ≥ 25 women with T2DM of reproductive age (18–45 years), this will provide a pooled sample of 750 women. Two local hospital diabetes pregnancy services responsible for the maternity care of 80–100 women with T2DM per year from this area will also participate, so that pregnancy outcomes can be studied.

It is difficult to predict how many women will be actively trying to conceive during the study period (this will be an important finding in itself), but we have estimated based on the number of annual pregnancies reported there will be 120–150 pregnancies in the participating boroughs: with 40–50 pregnancies in the women attending the PREPARED exposed practices. If we assume that women will be trying for pregnancies for 12 months prior to conception, then we can further estimate that > 100 women will be requiring PPC intervention with the remaining women (*n* =  > 650) requiring reproductive review and contraception.

There are high levels of deprivation and ethnic diversity in the local area, hence, establishing intervention reach into these communities is an important objective for the study as these groups of women form a major proportion of the target population.

### Data collection

The audit data (see Table 1) will be extracted from participating practices using an electronic template that we will install in the participating practices electronic record keeping system, the data collected will identify: women of child bearing age (< 45 years) attending appointments; reproductive assessments (activated on template); glycaemic control; changes to medications or contraception; PPC care-plan initiation; and PPC education or the dispensing of the integrated PREPARED self-help resources (leaflets and web-links). The pregnancy and pregnancy outcome data will be collected from the participating hospital audit systems which routinely collect these data for all women with diabetes as part of an ongoing national audit. The pregnancy booking data includes: glycaemic control; folic acid (5 mg) adherence; the prescription of any teratogenic therapies; and estimated pre-pregnancy weight. The birth outcome data includes: term and status of infant at birth including details of adverse outcomes; mode of birth; breast feeding status; maternal health/injury and depression; pregnancy complications; length of hospital stay; and neonatal care admissions. We will collect these data on all births during the observation period so we can compare outcomes in women from the intervention practices to unexposed practices. The hospital and practice level data will be extracted at baseline (12-month pre-PREPARED exposure) and then monthly to generate run charts monitoring process and outcome performance, with a full audit and review each quarter. The monthly data extractions will be continuous throughout the observation period (18 months). All data will be anonymised at source by hospital/practice administrators, practice codes will allow us to link them to the hospital data.

The unit of observation for determining performance within the audit will be the patient level care events of interest (i.e., a recorded occurrence of care actions or outcomes of relevance to the study performance outcomes). These data will be used to assess performance at two levels: the systemic level; and the practice level. The systemic level analysis will use the aggregated data from the participating practices detailing intervention impact on the activation of care processes and clinical outcomes, at the performance monitoring intervals to the study endpoint. The data will be used to populate run charts which will be updated monthly so that performance can be continuously assessed. The practice level data will enable us to consider inter-practice differences and the factors that may contribute to variations in performance between them. As the intention of this intervention is ultimately to improve local and national performance in achieving pre-pregnancy care targets and reduce adverse pregnancy outcomes, the primary estimate of impact will be at the systemic level. It should be noted that practice level feedback is also one of the intervention components; the practices will be able to see how well they are performing to either reaffirm progress and/or to stimulate further activity in achieving their performance goals.

The process evaluation data will involve qualitative and quantitative data collection using tools, and analytic models that have been designed following CAS [31], NPT [29, 30] and COM-B frameworks [28]. The qualitative data will include:

- Interviews with health professionals and support staff (*n* = 30), from the participating practices prior to, during (brief quarterly interviews) and at the end of the intervention period, to identify their experiences of the intervention including: any intervention barriers or enablers; how the intervention affected their ability to respond to women’s needs, monitor performance, anticipate needs and learnings; and ideas for intervention enhancement.
- Interviews with women with T2DM at the end of the observation period (*n* = 15), addressing their views on any reproductive information or support they have received and whether their understanding or views about pregnancy have changed.
- Interviews with up to 10 women who become pregnant during the observation period including women from practices that were and were not exposed to the intervention.
- We will ask practices who decline participation their reasons for this, and we will aim to conduct a short interview with staff from these practices (*n* = 5–10, depending on number of decliners).

The interviews will be conducted, either face-to-face or telephone (based on preference), and digitally recorded. Digital records will be transcribed verbatim in preparation for analysis using Framework Analysis [36]. This analysis will also identify emergent themes which can be integrated with the underpinning theoretical models (CAS, NPT and COM-B). A questionnaire will be sent to every practice in the participating boroughs to establish any changes to the practices that might impact on care delivery, to identify any contaminating or extraneous factors.

Quantitative data for the process evaluation will include:

- A report of the number and characteristics of eligible practices that accept or decline participation.
- A brief electronic questionnaire to all relevant health professionals (identified at practice sign-up) in participating practices to measure their baseline knowledge and beliefs in relation to reproductive health care in diabetes.
- A quarterly audit of intervention adherence based on recorded intervention utilisation as extracted from the intervention templates on the electronic records system.
- Exit questionnaires to all the health professionals and practice managers in the intervention practices, considering: NPT components [37, 38] following programme exposure; intervention satisfaction and utility; three strengths, weaknesses and areas for improving the programme; and a follow-up measure of health professional knowledge and beliefs in relation to reproductive care to assess learning.

The audit and questionnaire data will be analysed to provide insights on the acceptability, utilisation, reach and satisfaction of the programme. Intervention fidelity will be assessed from the audit data detailing the delivery of the different intervention components.

### Data integration and analysis

The data will provide ongoing information of intervention performance and implementation. The run charts and emergent findings of the process evaluation will be reviewed monthly with a full quarterly review by the research team and every 6 months by the advisory board to identify areas for refining or boosting intervention components to maximise the programme effects. The target performance for the intervention for the final observation will be achieving a minimum of 30% of women (from intervention exposed practices) achieving the following PPC objectives:

- HbA1c ≤ 48 mmol/mol (6.5%)
- Prescription of 5 mg folic acid pre-pregnancy
- Cessation of potentially teratogenic therapies

Practice level performance will also be monitored considering the proportion of women:

- Having a reproductive review and care plan instigated
- Engaged in the pre-pregnancy care pathway—referred to intermediate or hospital diabetes teams
- Commencing on folic acid 5 mg
- Instigated on contraception
- HbA1c testing and results

The extracted data will be imported into SPSS v26 and checked for accuracy (missing data or errors). The primary analysis will be based on collating the collective outcome and performance data from all the participating practices into run charts and performance metrics. In terms of PPC criteria, baseline performance will be established and then we will monitor performance prospectively (four-monthly intervals), to establish progression in performance as percentages to the final 30% target.

Hence, we will be able to provide a robust estimate of intervention effects and costs. We will also be able to compare the antenatal and pregnancy outcome data from the intervention practices to the non-participating practices to establish estimate differences in performance for the main performance measures and pregnancy outcomes. We will also consider practice level variations in process performance.

We will also report pregnancy interventions (initiation of additional hypoglycaemic agents) and birth outcomes (macrosomia, shoulder dystocia, caesarean rates, neonatal admissions, postnatal length of stay and other adverse outcomes); comparing practices exposed and not exposed to the PREPARED interventions. The process evaluation data will provide insights into cultural, interactional, and behavioural changes in relation to women and health professionals. These data will help us consider whether the intervention has changed the way care is delivered and how it is experienced by women with T2DM.

### Intervention optimisation

At the end of the study, we will be able to assess how each component of the PREPARED intervention was refined during the study and their relative contributions to PPC performance. Based on this analysis, together with the findings of the process evaluation, we will revise the PREPARED programme. The revised programme will be presented at a stakeholder event bringing together women with T2DM, health professionals (primary care, diabetes, and maternal health) and service administrators, managers, and commissioners.

### Health economics

We will conduct a cost consequences analysis of the costs and consequences of the improved PPC in women with T2DM based on the performance monitoring of the intervention. Costs and consequences of outcomes will be compared before intervention and after implementation. Outcome data will be drawn from analysis of performance on each of the three clinical objectives (HbA1c control, folic acid supplementation, cessation of teratogenic drugs) at baseline and final observation after optimisation. We will derive the cost of delivering the optimised intervention based on assessment of resource use required for each component. We anticipate the main costs to be GP and practice nurse time inputs; and we will monitor this as part of the process evaluation. Costs will be compared between exposed and unexposed women using generalised linear modelling (gamma family, identity link) as recommended to account for the highly skewed nature of cost data with bootstrapped confidence intervals [39]. We will conduct a systematic review of the extensive diabetes economic modelling literature for less frequent outcomes such stillbirth, pregnancy complications and neonatal disabilities. We will report the overall projected total cost of the PREPARED programme (including the impact on the costs of adverse health outcomes) and the impact on health outcomes in the form of a cost consequences analysis.

### Ethical considerations

We do not anticipate any major risk to the participants in context of this study. Informed written consent will be obtained from all participating women living with Type 2 diabetes, their partners, and the health professionals. Participants will be compensated for their involvement in this study. Research publications and data dissemination will not include identifiable data. UK- General Data Protection Regulation will apply to all aspects of the conduct of this study. An ethical approval review has been conducted and the study has been approved by the Health Research Authority in the United Kingdom, Research Ethics Committee reference: 21/LO/0823.

## Discussion

This paper has outlined the protocol to evaluate and optimise a complex intervention designed to improve PPC uptake and pregnancy outcomes in women with T2DM. The success of the PREPARED programme is important as currently very few women are receiving appropriate PPC contributing to the high levels of adverse pregnancy outcomes in this population. The novelty of this programme compared to previous intervention studies [20–23] is in its focus exclusively on women with T2DM in primary care. An additional potential advantage for our study, is in the methods we propose. As the intervention is complex targeting multiple pathways, we are using a method that addresses the potential interaction between the behaviours of health professionals and the care system in order to allow us to study how well the intervention components are performing so we can optimise them and also study how best to implement the PREPARED programme. There is an emerging recognition that linear models of intervention development and evaluation (such as traditional cluster trials) may not be optimal in studying complex interventions efficiently, particularly when multiple systematic and behavioural factors can mediate outcome assessment [40]. Hence, we hope that by using these methods we will be able to produce an effective strategy for increasing PPC. The study will also contribute to the growing number of studies using approaches grounded in CAS principles [41].

### Study outputs and impact

Our study is addressing a significant and growing problem that is associated with adverse pregnancy outcomes that are very damaging to mothers and their babies. The lack of PPC contributes considerably to pregnancy costs. Hence, if we achieve the performance target for the intervention, we will have an implementable programme of intervention which could significantly improve pregnancy outcomes; reduce impacts on women’s physical and mental health; and reduce National Health Service (NHS) treatment costs. The study has also been designed to yield important insights into potential implementation barriers and how these can be overcome. Given the failure of previous interventions to make significant improvements in meeting NICE PPC criteria [17], having these insights will greatly enhance the impact of our work. This study will provide:

- New knowledge (and transferable resources) on how to improve women’s understanding of diabetes and pregnancy and what they can do to reduce the risks for themselves and their babies.
- A care pathway to support women and health professionals to identify what women need at different points in their reproductive cycle, and how and where to access the resources they need (integration).
- The development of decision support tools for health professionals to identify risks and to help them work productively with women to enhance the reproductive care they receive.
- Strategies to promote a wider community awareness of the reproductive needs of women living with T2DM that address potential cultural barriers to engaging with pre-pregnancy care.
- System level strategies to promote the visibility of women of reproductive age with T2DM in the health system.
- Evidence of costs and consequences of intervention and commissioning principles.

Finally, as we have been working with women living with T2DM throughout our research, it is important to recognise their contribution to the study. We have a strong patient involvement group of women with T2DM who contributed to our previous studies in developing the PREPARED programme. In this study we have a patient co-applicant who has experienced pregnancies with T2DM, and she will jointly chair the project advisory board (she is one of the authors of this paper OA). She with our other patient representatives (*n* = 4) have input on reviewing performance and optimising the intervention. We are also working closely with primary care, diabetes, and pregnancy services to ensure that what emerges from the study will be acceptable to care systems across the UK.

## Data Availability

The datasets generated and/or analysed during this study will not be publicly available but are available from the corresponding author on reasonable request.

## Abbreviations

BCW: Behaviour Change Wheel
CAS: Complex Adaptive Systems
CEMACH: Confidential enquiry maternal and child health
COM-B: Capability, Opportunity, Motivation to perform a Behaviour
GP: General Practitioner
HbA1c: Glycosylated haemoglobin
NHS: National Health Service
NICE: National Institute for Clinical excellence
NPT: Normalisation Process Theory
PPC: Pre-pregnancy care
PREPARED: Pre-pregnancy care for women with Type 2 diabetes
T1DM: Type 1 diabetes mellitus
T2DM: Type 2 diabetes mellitus
